# Growth factors in the regulation of reparative response in the presence of peritoneal damage

**DOI:** 10.1515/pp-2020-0114

**Published:** 2020-11-02

**Authors:** Irina A. Shurygina, Мichael G. Shurygin, Lubov V. Rodionova, Nataliya I. Ayushinova

**Affiliations:** Irkutsk Scientific Center of Surgery and Traumatology, Irkutsk, Russia; Pharmasyntez, Irkutsk, Russia

**Keywords:** growth factor, laparotomy, PCR, peritoneal adhesion

## Abstract

**Objectives:**

To study the expression of growth factors in the regulation of tissue repair after peritoneal damage tissue response to peritoneal damage.

**Methods:**

Experimental study in 35 male Wistar rats determining the evolution over time of the tissue response to aseptic peritoneal damage. A standardized bowel and peritoneal lesions were created in the right lower quadrant by laparotomy. Then, tissular expression of growth factors was evaluated by multiplex polymerase chain reaction at seven timepoints between 6 h and 30 days, postoperatively.

**Results:**

Tissular responses of granulocyte-stimulating factors (Csf2, Csf3), connective tissue growth factor (Ctgf), epidermal growth factors and receptor (Egf, Egfr), fibroblast growth factors (Fgf2, 7 and 10), heparin binding EGF-like growth factor (Hbegf), hepatocyte growth factor (Hgf), insulin-like growth factor-1 (Igf1), mitogenic transforming growth factors (Tgfa, Tgfb1, Tgfbr3), and vascular endothelial growth factor A (Vegfa) were biphasic with a first expression peak at day 3, followed by a more pronounced peak at day 14.

**Conclusions:**

We observed a long-lasting, widespread response of tissular growth factors for at least two weeks after peritoneal damage. To be clinically effective, the prophylaxis of postoperative adhesions might be needed for an extended period of time.

## Introduction

In recent decades, growth factors have attracted a lot of attention in the study of the pathogenesis of various diseases and conditions because they play a crucial role in the processes of growth, development, and maintenance of cell populations. Growth factors are biologically active polypeptides that function as regulatory signals controlling cell proliferation and differentiation, and they promote cell survival [1], [2].

Most growth factors are multipotent and can affect various cell types. Although their names often reflect specific cellular types, their action might not be limited to this cell population. For example, vascular endothelial growth factor (VEGF) stimulates not only vascular growth. Still, it is also involved in the proliferation, migration, differentiation, and mobility of fibroblasts, both in physiological and reparative regeneration processes [3], [4], [5]. Despite the affinity for vascular endothelial cells, the level of VEGF significantly affects collagen production during the development of postinfarction cardiosclerosis [6]. Similarly, fibroblast growth factor 2 (FGF2) can act as an activator of angiogenesis [7], [8].

The general role of growth factors in the regulation of physiological and reparative regeneration processes response has been extensively documented. FGF and VEGF are the master regulators of connective tissue formation; they activate the repair in the damaged tissue by stimulating fibroblast migration and the growth of granulation tissue. However, some peculiarities are depending on the organ localization of its formation and tissue environment [9].

Growth factors play an essential role in repairing the peritoneal damage and have a significant impact on the development of adhesions in the abdominal cavity, for example, after surgery. Transforming growth factor-beta (TGF-β) expression has been documented from the third postoperative day and increases gradually until day 14 [10]. Conversely, blocking TGF-β1 reduces the development of peritoneal fibrosis [11], [12]. A coexpression of connective tissue growth factor (CTGF) was observed with increasing fibrosis and angiogenesis in a peritoneal adhesion model in the rodent, with a peak between postoperative days 6 and 15. Thus, CTGF is another critical molecule in fibrous adhesive disease and might be a target for future adhesion prevention [13], [14]. Intraperitoneal administration of Bevacizumab, a recombinant monoclonal antibody blocking VEGF, decreased the intensity of peritoneal adhesions in the rat model [15]. Less peritoneal adhesions developed when insulin-like growth factor I (IGF1) was blocked by administrating IGF-binding protein 4 intraperitoneally [16]. Local application of epidermal growth factor (EGF) as a gelatin film also inhibited the formation of peritoneal adhesions [17].

However, the expression of growth factors in the formation of adhesions after peritoneal damage has received relatively little attention. The purpose of this study was to investigate the response of the peritoneal tissue to injury by examining the expression of a large panel of growth factors during the development of the adhesive process.

## Materials and methods

### Study design

In an experimental study in 35 male Wistar rats, we determined the tissue response to aseptic peritoneal damage over time. A standardized bowel and peritoneal lesion were created in the right lower quadrant by laparotomy. Then, tissular expression of growth factors was evaluated by multiplex polymerase chain reaction (PCR) at seven timepoints between 6 h and 30 days, postoperatively.

### Regulatory background

The study protocol was approved by the Institutional Review Board of the Irkutsk Scientific Center of Surgery and Traumatology, Irkutsk, Russia. The experiments were carried out under the rules of animal welfare proposed by the International Guidelines of the Association for Assessment and Accreditation of Laboratory Animal Care International (AAALAC).

### Animals and experiment

Thirty-five, nine-month-old male Wistar rats weighing 220–250 g were purchased from a commercial provider. The animals were narcotized using ketamine 50 mg/kg, droperidol 2.5 mg/kg and atropine 0.4 mg/kg.

**Figure 1:**
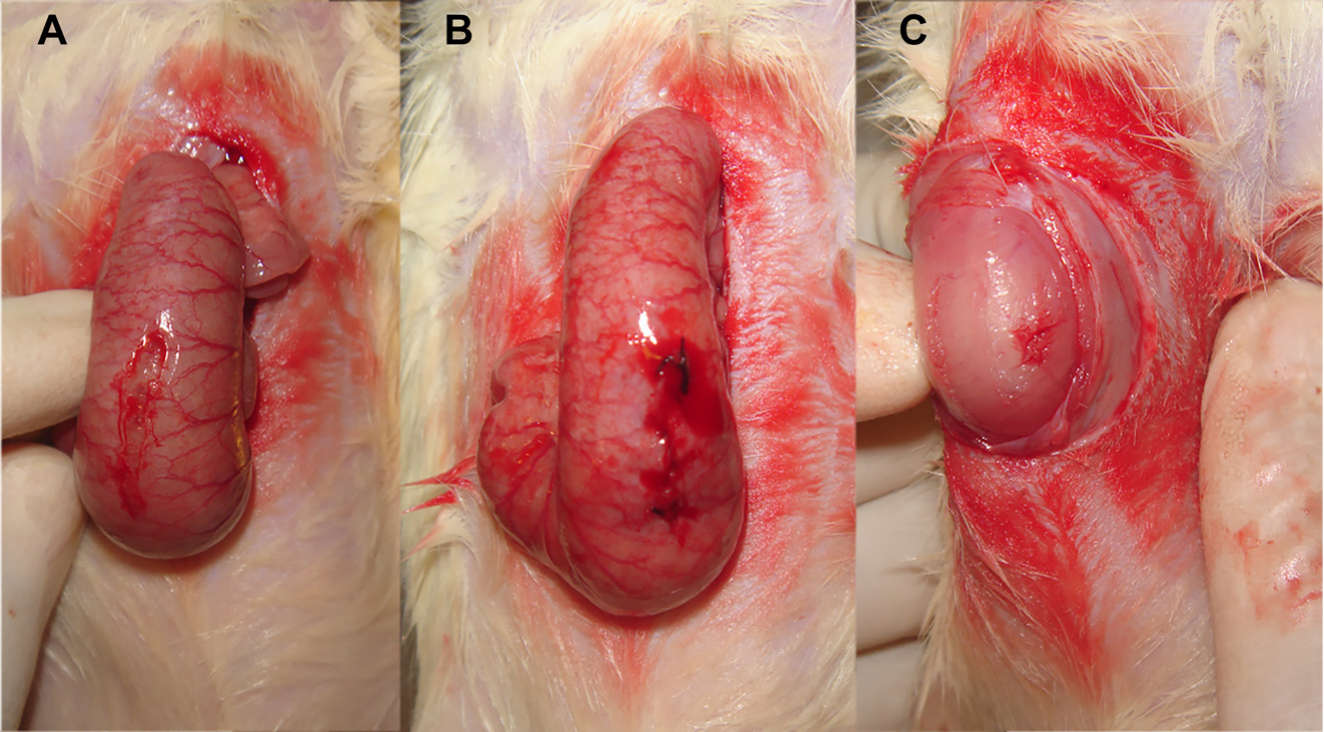
Surgical, standardized peritoneal lesion for inducing an aseptic inflammatory response. (A) Incision of the seromuscular layers of the cecum. (B) Suturing the incision. (C) Scarification of the peritoneum in the right paracolic gutter.

After median laparotomy, the serous-muscular layer of the cecum was opened with a length of 1 cm, followed by suturing of the wound with a self-twisting continuous suture with a step of 2 mm. The needle was inserted laterally to the incision, at a distance of 1 mm from the edge. The incision was immediately sutured with atraumatic VICRYL® (polyglactin 910) stitches (needle RB-1 Plus, 1/2, 17 mm). Then, the peritoneum was scarified in the right paracolic gutter over a surface of 1.5 × 1.5 cm^2^, by scratching the surface over 2–3 mm with a scalpel tip in the longitudinal and transverse directions, as described previously [18], [19] (Figure 1).

The animals were euthanatized at seven time points ranging (6 h, 12 h, 1 day, 3 days, 7 days, 14 days, and 30 days) postoperatively, and peritoneal tissues were taken for further analysis. The serous-muscular layer of the cecum in intact animals served as a control (n=5).

### Biospecimen collection

Biopsies of 2 × 2 mm^2^ were taken under sterile conditions from the damaged area of the cecum. Biopsies were plunged into RNAlater solution (Cat # 7020, Ambion, Canada) for 12 h, and then frozen at −20 °C until further analysis.

### Preanalytical sample preparation

The biopsies were broken up by grinding in a mortar with liquid nitrogen to prevent thawing of the tissue. Samples were homogenized by passing the solution 10-fold through the needle of a 5-mL syringe until complete homogeneity and transparency were obtained. In order to avoid microbial or RNase contamination, preanalytical sample preparation was carried out under sterile conditions.

### RNA extraction

RNeasy Mini Kit was used (Qiagen GmbH, Germany, Cat. No. 74104) for RNA extraction. Total RNA was extracted from tissues according to the manufacturer’s instructions. The concentration of total RNA was determined using a spectrophotometer (Shimadzu, Japan). To additional purify RNA preparations from DNase commercial kit “Rnase-Free DNase Set” (Qiagen GmbH, Германия, Cat. No. 79254) was used.

### Reverse transcription

cDNA was generated from 300 ng total RNA using a commercial kit (cDNA – “RT^2^ First Strand Kit”, Qiagen GmbH, Германия, Cat. No. 330401), according to the instructions of the manufacturer. The possible genomic DNA impurities were removed during the procedure.

### Real-time PCR

Gene expression was analyzed by real-time PCR (CFX96, Bio Rad, USA), using a customized array (PCR – RT2-Profiler™ Array Rat Wound Healing Kit, Qiagen, Germany). A list of genes is given in Table 1. We used a set of oligonucleotides RT2 SYBR Green qPCR Mastermix (Cat. No. 330503, Qiagen, Germany). Housekeeping genes and controls for genomic contamination and reverse transcription were included in each run. Relative fold difference in gene expression for Wound Healing-associated genes was calculated using the 2^−ΔΔCT^ method compared with intact animal controls.

**Table 1:** A multidimensional gene panel of growth factors was selected to monitor over a longer period time (30 days) several aspects of the wound repair process after peritoneal injury.

Gene symbol	Full name	GenBank	Unigene	PCR primers
Csf2	Colony stimulating factor 2 (granulocyte-macrophage)	XM_340799	Rn.44285	PPR49732A
Csf3	Colony stimulating factor 3 (granulocyte)	NM_017104	Rn.53973	PPR50733A
Ctgf	Connective tissue growth factor	NM_022266	Rn.17145	PPR46426B
Egf	Epidermal growth factor	NM_012842	Rn.6075	PPR43509B
Egfr	Epidermal growth factor receptor	NM_031507	Rn.37227	PPR48960A
Fgf10	Fibroblast growth factor 10	NM_012951	Rn.44439	PPR06632A
Fgf2	Fibroblast growth factor 2	NM_019305	Rn.31808	PPR06641B
Fgf7	Fibroblast growth factor 7	NM_022182	Rn.98842	PPR06649A
Hbegf	Heparin-binding EGF-like growth factor	NM_012945	Rn.10148	PPR44696B
Hgf	Hepatocyte growth factor	NM_017017	Rn.10468	PPR44869B
Igf1	Insulin-like growth factor 1	NM_178866	Rn.6282	PPR06664F
Tgfa	Transforming growth factor alpha	NM_012671	Rn.9952	PPR06486A
Tgfb1	Transforming growth factor, beta 1	NM_021578	Rn.40136	PPR06430B
Tgfbr3	Transforming growth factor, beta receptor III	NM_017256	Rn.9953	PPR06487A
Vegfa	Vascular endothelial growth factor A	NM_031836	Rn.1923	PPR06748C

### Statistical analysis

Data are presented as mean and CI 5–95%. Statistical analysis of gene expression was performed using the software provided with the kit (http://www.qiagen.com/Products/Genes and Pathways/Data Analysis Center Overview Page / RT2 Profiler PCR Arrays Data Analysis Center). This software takes into account multiple comparison testing.

## Results

In this experimental study, we investigated the expression of a large panel of growth factors involved in wound healing. These included granulocyte-stimulating factors (Csf2, Csf3), connective tissue growth factor (Ctgf), epidermal growth factors and receptor (Egf, Egfr), fibroblast growth factors (Fgf2, 7 and 10), heparin binding EGF-like growth factor (Hbegf), hepatocyte growth factor (Hgf), insulin-like growth factor-1 (Igf1), mitogenic transforming growth factors (Tgfa, Tgfb1, Tgfbr3), and vascular endothelial growth factor A (Vegfa).

**Figure 2:**
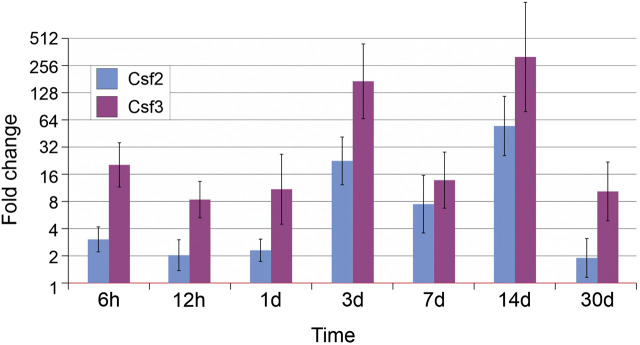
Expression levels of granulocyte-stimulating factors Csf2 and Csf3.

First, we examined the granulocyte and macrophage recruitment in the damaged area. As shown in Figure 2, we observed an early, moderate increase in the expression of Csf2 (3.04-fold) and Csf3 (20.02-fold) already 6 h after peritoneal injury, as compared to intact animals. The expression reached a first peak at postoperative day (Csf2: 22.94-fold, Csf3: 169.29 times) and then decreased by day 7. A second, more pronounced expression peak was observed by day 14 (Csf2: 55.20-fold; Csf3; 312.2-fold). At the end of the observation period (postoperative day 30), the expression levels decreased but were not back to the normal (Csf3: 10.51-fold increase).

A similar expression profile over time with an early and a later expression peak was also observed for connective and epidermal growth factors. Expression of connective tissue growth factor (Ctgf), e exceeded the indices of intact animals on postoperative 3 and 14 by a factor 3.53 and 5.80, respectively (Supplementary Material, Figure A). Two similar peaks of gene expression were noted for Egf and Egfr (Supplementary Material, Figure B). Whereas expression of Egf on days 3 and 14 were almost identical, the level of Egfr on day 14 was more than twofold higher than on day 3, confirming the presence and the intensity of the second reaction phase to peritoneal injury after two weeks.

**Figure 3:**
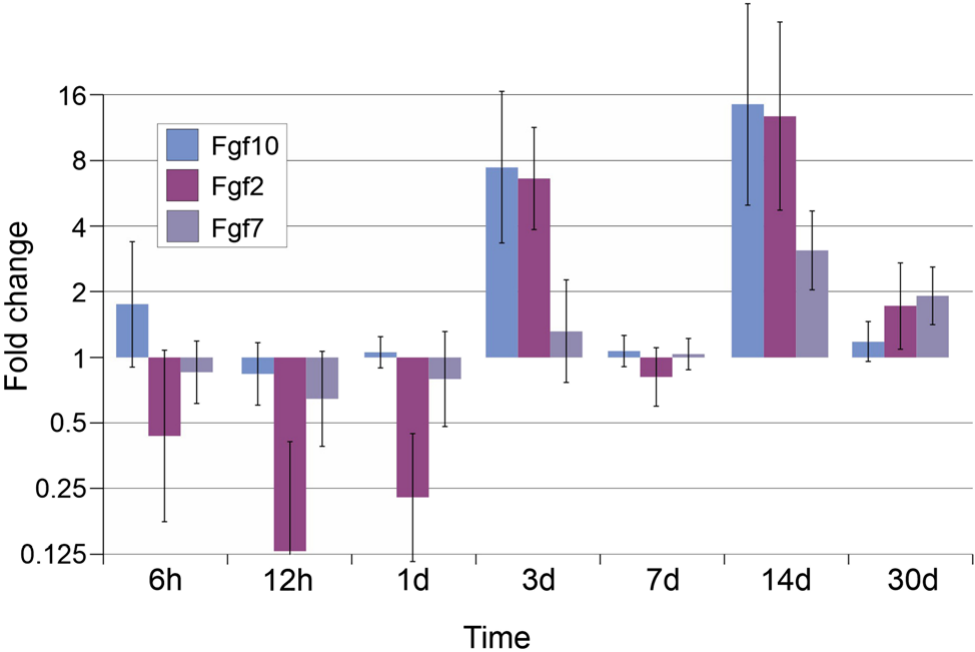
Expression levels of fibroblast growth factors (Fgf2, 7, and 10).

**Figure 4:**
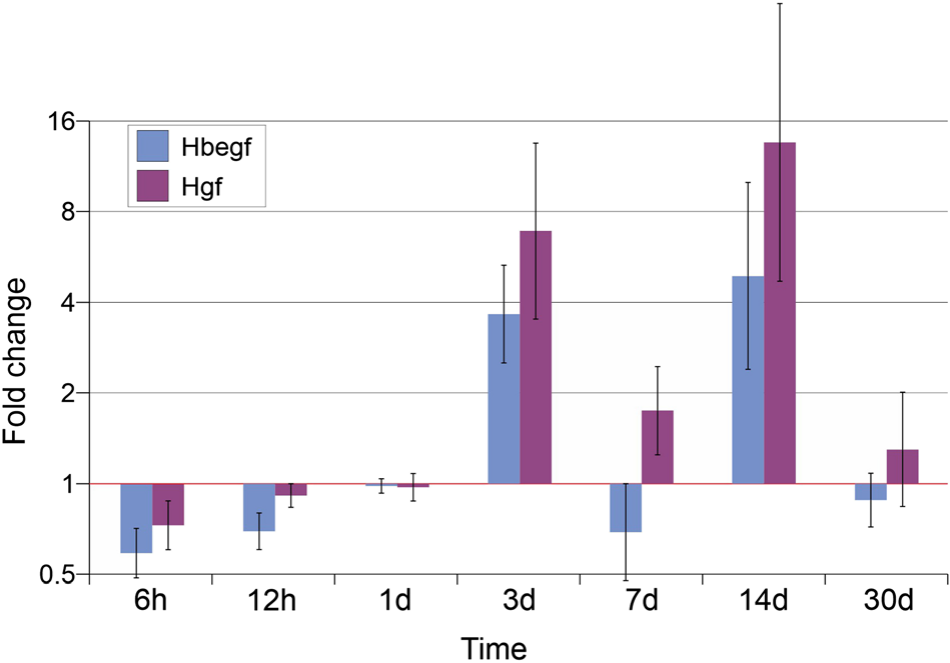
Expression levels of heparin binding EGF-like growth factor (Hbegf) and hepatocyte growth factor (Hgf).

When expression of fibroblast growth factors (Fgf2, 7, and 10), was examined, two activity peaks were also found on days 3 and 14 for Fgf2 and 10, while expression of Fgf7 was characterized by a single peak on day 14 (threefold increase as compared to intact animals) (Figure 3). The expression of the heparin-related epidermal-like growth factor Hbegf and of hepatocyte growth factor Hgf also increased on days 3 and 14 with a maximum by day 14 (Figure 4).

We also examined neoangiogenesis in the wound. Unexpectedly, the increase of VEGF expression during the reparative response was relatively moderate. Here again, two activity peaks were seen on days 3 and 14, but the maximum activity increased only by 2.74-fold on day 14 as compared to intact animals. A long-lasting increase of insulin-like growth factor 1 was noted on days 14 and 30 (Supplementary Material, Figure C). Expression levels of Tgfb1, as well as of Tgfbr3, increased significantly only on day 14, with no early response observed on day 3. The increase of Tgfa gene expression was minimal (Supplementary Material, Figure D).

## Discussion

Although postoperative adhesions remain an unsolved challenge in modern surgery, expression of growth factors in the formation of adhesions after peritoneal damage has received little attention. The purpose of this study was to investigate the response of the peritoneal tissue to injury by examining the expression of growth factors over time during the development of the adhesive process. For this purpose, to induce an aseptic inflammatory response, we created standardized peritoneal damage in rodents and determined the expression of a large panel of growth factors. We included granulocyte-stimulating factors (Csf2, Csf3), connective tissue growth factor (Ctgf), epidermal growth factors and receptor (Egf, Egfr), fibroblast growth factors (Fgf2, 7 and 10), heparin binding EGF-like growth factor (Hbegf), hepatocyte growth factor (Hgf), insulin-like growth factor-1 (Igf1), mitogenic transforming growth factors (Tgfa, Tgfb1, Tgfbr3), and vascular endothelial growth factor A (Vegfa) in our analysis. The hope was to detect common patterns of expression of these growth factors over the wound healing process.

Our main finding is that tissular expression of growth factors during peritoneal wound repair is a biphasic process, with an early phase culminating on day 3 after injury, and a late phase reaching its maximum two weeks after this injury. These results are in line with the expression of proinflammatory cytokines in the abdominal cavity [20], [21]. Notably, the second peak at day 14 was usually more pronounced. Whereas the first activity peak on day 3 after the peritoneal injury is easily explained by the need for the growth of granulation tissue in the damaged area [22], the presence and the intensity of the peak on day 14 were not expected and now deserve closer attention.

Peritoneal adhesions frequently develop after abdominal surgery and can cause a significant burden, such as chronic abdominal pain, small bowel obstruction, or infertility. The etiology of peritoneal adhesions is multifactorial and involves a dysbalance between coagulation, inflammation, and fibrinolysis. Cellular components such as monocytes, macrophages, fibroblasts, and mesothelial cells are involved in this process. Moreover, growth factors and secreted signaling molecules play a key role in peritoneal adhesions development [23]. Peritoneal mesothelial cells (PMC) maintain a balance between procoagulant and fibrinolytic activation by producing a whole range of corresponding regulators. PMC defects and lesions cause an unbalance between procoagulant and fibrinolytic properties resulting in the formation of fibrin bands and, later on, of peritoneal adhesions [24].

Prevention of postoperative adhesions is an active area of research. Various drugs and methods such as icodextrin, barriers, and others have been clinically tested, but their effectiveness is debated [23]. Against this framework, our results indicate a long-lasting involvement of growth factors in the reparative process after peritoneal damage.

We make the hypothesis that a single intraoperative administration of antiadhesive agents might be insufficient to prevent the formation of postoperative adhesions. Such a single administration would have an effect on the first active phase of growth stimulation immediately after surgery but is unlikely to influence the second growth phase we demonstrated to last at least two weeks postoperatively. This observation calls indeed for long-term preventive strategies, with demonstrated target effect during at least two weeks after surgery. Our hypothesis is supported by the fact that gels and other biomaterials appear to be the most effective adhesion prevention agents for use during gynecological surgery [25]. Gel formulation guarantees a slow and long-lasting release of the active agents indeed into the peritoneal cavity and the intra-abdominal postoperative wounds.

Future research should evaluate the ability of novel preventing approaches to reduce the expression of growth factors over a more extended period. We propose to use our established experimental model of peritoneal injury and the gene panel above as a surrogate marker of efficacy. Such an approach might be useful for speeding up the validation of novel prophylactic strategies against postoperative adhesions.
