# Supplementary figures and images for: Growth factors in the regulation of reparative response in the presence of peritoneal damage
Source: Pleura Peritoneum. 2020 Nov 2;5(4):20200114. doi: 10.1515/pp-2020-0114 (PMC7823155; doi:10.1515/pp-2020-0114)

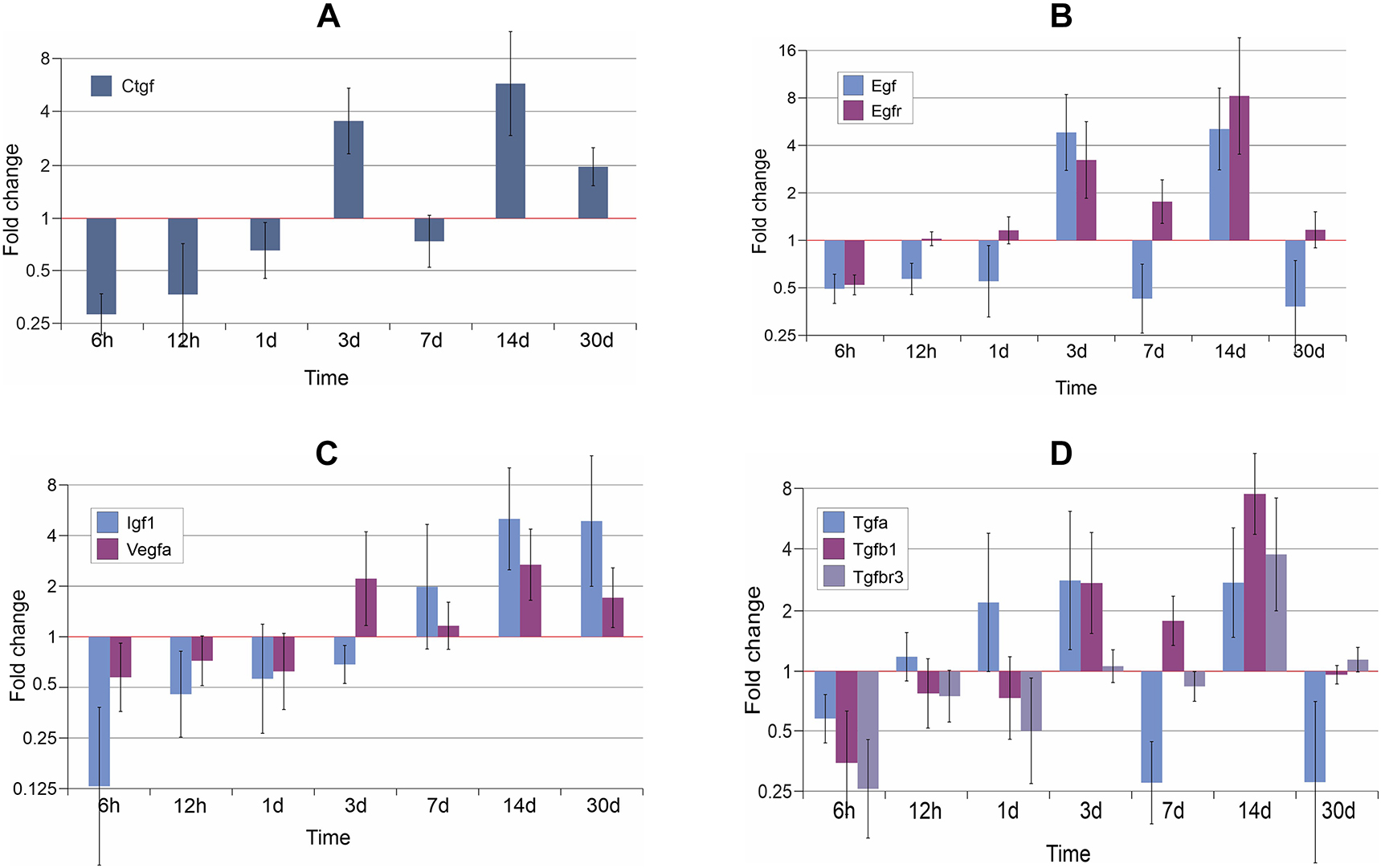

Supplement: Supplementary file 1 [file pp-05-20200114-s001.tif]
